# Influence of TMPRSS6 genotype on iron status parameters in stable COPD patients

**DOI:** 10.5937/jomb0-52996

**Published:** 2025-01-24

**Authors:** Marko Trtica, Ivana Novaković, Violeta Dopsaj, Branislava Milenković, Jelena Janković, Sanja Dimić-Janjić, Vesna Dopuđa-Pantić, Jelena Martinović, Snežana Jovičić

**Affiliations:** 1 University of Belgrade, Faculty of Pharmacy, Department of Medical Biochemistry, Belgrade; 2 University of Belgrade, Faculty of Medicine, Institute of Human Genetics, Belgrade; 3 University of Belgrade, Faculty of Medicine, Belgrade; 4 University Clinical Centre of Serbia, Clinic for Pulmonology, Belgrade; 5 Zvezdara Clinical Hospital Center, Clinical Department of Pulmonology and Allergology with Immunology, Belgrade; 6 Sante Biohemija, Belgrade

**Keywords:** B12 deficiency, COPD, erythropoietin, hepcidin, iron status parameters, TMPRSS6 gene polymorphism, deficijencija vitamina B12, eritropoetin, hepcidin, HOBP, parametri statusa gvožđa, polimorfizam gena TMPRSS6

## Abstract

**Background:**

The SNP rs855791 has been linked to increased hepcidin levels, variations in serum iron, transferrin saturation and red blood cell indices. Our goal was to determine the prevalence of this polymorphism among COPD patients and to assess its impact on iron status parameters in patients with stable COPD.

**Methods:**

We analysed iron status parameters and genetic data from 29 COPD patients with wild-type genotype (WT group) and 65 COPD patients with either homozygous or heterozygous genotype (HH group). Additionally, the prevalence of SNP rs855791 was assessed in 192 volunteers.

**Results:**

The frequency distribution of SNP rs855791 was comparable between the COPD patients and control subjects (p=0.791). Iron status parameters were within their respective reference values and showed neither statistically nor clinically significant difference between the WT and HH group of COPD patients. However, after excluding patients with (sub)clinical vitamin B12 deficiency and/or hypoxemia, WT group of patients exhibited significantly lower erythropoietin levels (p=0.015). The area under the curve for erythropoietin was 0.688 (95% CI: 0.545-0.830, p=0.015), with an optimal cut-off of 9.74, sensitivity of 61.2% (95% CI: 58.1-64.3) and specificity of 65.0% (95% CI: 61.8-68.3).

**Conclusions:**

Iron status parameters do not differ between WT and HH groups of stable COPD patients. Statistical but not clinical difference in EPO levels was observed in a subgroup of patients. In addition to promoting erythropoiesis, EPO may regulate hepcidin levels and thus influence the development of iron deficiency and/or anaemia. Also, EPO's direct effect on immune cells and down-regulation of inflammatory reactions should be considered in this context.

## Abstract (sr)

**Uvod:**

Polimorfizam rs855791 povezan je s povišenim vrednostima hepcidina i promenama u vrednostima serumskog gvožđa, saturacije transferina i eritrocitnim indeksima. Cilj istraživanja bio je da se odredi zastupljenost ovog polimorfizma kod pacijenata sa HOBP, kao i da se ispita njegov uticaj na vrednosti parametara statusa gvožđa kod pacijenata sa stabilnim oblikom HOBP

**Metode:**

Parametri statusa gvožđa i zastupljenost genotipova su ispitani kod 29 HOBP pacijenata s wild-type tip varijantom (WT grupa) i 65 HOBP pacijenata s homozigotnom ili heterozigotnom varijantom (HH grupa). Zastupljenost genotipova određena je i kod 192 ispitanika kontrolne grupe.

**Rezultati:**

Zastupljenosti polimorfizma rs855791 kod pacijenata s HOBP i ispitanika kontrolne grupe je bila uporediva (p=0.791). Parametri statusa gvožđa su bili u okvirima svojih referentnih vrednosti i nije bilo ni statistički ni klinički značajne razlike između WT i HH grupe pacijenata s HOBP Nakon isključenja pacijenata sa (sub)kliničkim deficitom vitamina B12 i/ili hipoksijom, dobijene su statistički značajno niže vrednosti eritropoetina kod ispitanika WT grupe (p=0.015). Površina ispod krive (AUC) iznosila je 0.688 (95% CI: 0.545-0.830, p=0.015). Određena je optimalna granična vrednost za eritropoetin od 9,74 mIU/mL sa osetljivošću od 61.2% (95% CI: 58.1-64.3) i specifičnošću od 65.0% (95% CI: 61.8-68.3).

**Zaključak:**

Kod pacijenata sa stabilnim oblikom HOBP parametri statusa gvožđa se ne razlikuju između pacijenata sa wild-type i pacijenata s homozigotnom ili heterozigotnom varijantom. Statistički, ali ne i klinički, značajna razlika u vrednosti eritropoetina je dobijena u podgrupi pacijenata. Uz svoju poznatu ulogu u eritropoezi, eritropoetin bi mogao da utiče na vrednosti hepcidina i tako spreči nastanak deficijencije gvožđa i/ili anemije. Takođe, u ovom kontekstu treba razmotriti i direktni efekat eritropoetina na ćelije imunog sistema i nishodnu regulaciju inflamacije.

## Introduction

Chronic obstructive pulmonary disease (COPD) is a preventable and treatable disease. It is characterised by airflow limitation as well as by significant systemic manifestations which add to general severity of the condition. A prominent feature of COPD is enhanced inflammatory response to harmful particles or gases, particularly cigarette smoke. Significant increase in circulating cytokines, acute-phase proteins and inflammatory cell influxes is well documented in the clinical setting of COPD [1] [2] [3] [4] [5].

The results of several genome-wide association studies (GWAS) have highlighted the impact of abnormal iron metabolism in COPD [5] [6] [7] [8]. Although COPD has a well-known association with polycythaemia, anaemia is also increasingly recognised as a significant comorbidity, affecting many patients and adversely impacting their overall prognosis [1] [2] [9] [10] [11] [12].

The main origin of iron deficiency (ID) and anaemia in COPD is chronic inflammation, though factors such as nutritional deficiencies, therapeutical approaches, comorbidities also contribute to disturbances in iron homeostasis [9]. Certain evidence suggests that smoking either alone or together with COPD may increase the permeability of pulmonary vessels, leading to the spill-over of in ammatory mediators from the pulmonary to the systemic compartment which could induce raise in hepcidin concentration [11]. Cigarette smoke was shown to increase haemoglobin levels and may affect the incidence and recognition of anaemia by causing polycythaemia and by having negative effect on haematopoiesis. Also, both current and former smokers are shown to have increased levels of serum ferritin [5]. Iron content as well as iron-binding molecules like ferritin, lactoferrin, lipocalin are all elevated in the lungs, alveolar macrophages, bronchoalveolar lavage fluid of COPD patients [5].

The identification of hepcidin as a key regulator of iron homeostasis has provided us with new insights regarding iron deficiency and overload and helped in recognition of those conditions [13]. Hepcidin synthesis is regulated by multiple stimuli including iron levels, in ammation, hypoxia, anaemia, and erythroid factors. The complexity of its synthesis may be described through conflicting effects of inflammation and hypoxia, with important influence of dietary habits, applied medicinal methods and other clinically relevant circumstances [9] [11] [14].

Matriptase-2 (MT2) is a protease encoded by transmembrane protease serine 6 (TMPRSS6) gene. It is recognized as a key negative regulator of the receptor complex responsible for hepcidin transcription. Current knowledge suggests that MT2 functions as an allosteric inhibitor of the receptor but also as a protease which degrades certain elements of the receptor complex. Consequently, hepcidin transcription suppression occurs which promotes iron absorption as well as its release from the stores, thereby increasing iron availability [15] [16].

Genome-wide association studies focused on examining genetic determinants of important haematological traits and iron status have determined genetic variants in TMPRSS6 that are linked with changes in serum iron, transferrin saturation, volume of red blood cells and haemoglobin concentration [16] [17] [18] [19]. These TMPRSS6 genetic variants can result in a range of conditions, with iron-refractory iron deficiency anaemia (IRIDA) representing the most severe end of this spectrum [17].

The single nucleotide polymorphism (SNP) rs855791 results in a nonsynonymous substitution of alanine (A) with valine (V) at the position 736 (A736V) in the catalytic domain of TMPRSS6. According to a human database, valine is considered the »wild-type« amino acid at the 736 position. However, comparative analysis shows that alanine is the ancestral amino acid, as it is conserved across all species with annotated MT2 sequences. This suggests that the MT2 variant A736V, which increases hepcidin production and inhibits iron absorption, represents a more recent evolutionary development [16]. The missense A736V variant (TMPRSS6 rs855791) is the most frequently reported SNP linked to iron deficiency among non-African populations [17]. It is associated with iron deficiency anaemia and IRIDA, characterised by increased hepcidin levels, decreased iron, and reduced haemoglobin indices [16] [17].

Nai et al. [16] demonstrated that rs855791 is a functional variant of TMPRSS6 and that it affectsexpression of hepcidin in normal individuals. This implies that rs855791 impacts alterations in hepcidin levels in response to increases of circulating and total body iron thereby influencing iron parameters and, indirectly, erythropoiesis. Additionally, the p.A736V polymorphism has been described in the clinical context of iron overload in hereditary hemochromatosis and non-alcoholic fatty liver disease [20]. Nevertheless, it is unclear whether the A736V variant impacts iron homeostasis amid chronic inflammation such as that seen in COPD.

Iron deficiency and anaemia are shown to have serious and even deleterious clinical consequences in COPD patients including decreased respiratory function, pronounced symptoms with increased morbidity and mortality [1] [21]. TMPRSS6 rs855791 polymorphism is associated with inappropriately normal or high hepcidin levels, which implies that oral iron is ineffective and parenteral administration is necessary to achieve at least a partial response [22]. New therapeutic options include managing iron levels with intravenous iron therapy and the potential availability of hepcidin antagonists [21]. Personalised iron therapy approach is therefore necessary to avoid delays or unnecessary/harmful treatments [22].

This study aimed to determine the prevalence of SNP rs855791 TMPRSS6 A736V polymorphism in patients with COPD. Furthermore, we aimed to evaluate whether this polymorphism which regulates hepcidin transcription impacts levels of hepcidin and other indicators of iron status in patients with stable COPD.

## Materials and methods

### Participants

A total of 107 patients with confirmed COPD attending a routine check-up were recruited from two healthcare facilities from Belgrade, Serbia – the Clinic of Pulmonology, the University Clinical Centre of Serbia and the Clinical Department of Pulmo nology and Allergology with Immunology, Zvezdara Clinical Hospital Centre.

COPD diagnosis followed the Global Initiative for Chronic Obstructive Lung Disease (GOLD) guidelines, defined by a ratio of post bronchodilator forced expiratory volume in the first second (FEV_1_) and forced vital capacity (FVC): FEV_1_/FVC <0.7 [23].

The control group comprised of 192 healthy adults recruited from the Primary Healthcare Centre Rakovica, Belgrade, Serbia. They did not have any respiratory disease including COPD, and also did not meet defined exclusion conditions. The study adhered to the Declaration of Helsinki and received approval from the ethics committees of all three healthcare centres (reference numbers 715/7, 260319 and DZR170619/1 respectively). All participants gave written informed consent.

Clinicians performed a routine clinical evaluation of all 107 COPD patients: 94 patients (87.9%) were diagnosed with stable COPD while acute worsening of the disease (AECOPD) was discovered in 13 patients (12.1%) hence they were excluded from the study. Applied definition of stable COPD was: no alteration in respiratory status in the previous month that would necessitate modification in treatment or the use of antibiotics and/or systemic steroids [24] [25]. An acute exacerbation of COPD was defined as a significant change of at least one of the respiratory symptoms e.g., increased sputum volume or purulence, an intensified dyspnoea or cough requiring usage of antibiotics and/or systemic corticosteroids or emergency room visit and/or hospitalisation [26] [27].

We excluded patients with any pulmonary disease other than COPD or who had AECOPD in theprevious month. Furthermore, we excluded patients with sepsis or any other autoimmune disorder, or with malignancy in the previous five years. Treatments with supplements of iron, vitamin B12 or folate, oral or parenteral corticosteroids as well as with erythropoietin in the previous three months were also excluding criteria. Existing anaemia as per World Health Organization definition [28], diagnosed haemoglobin opathy and/or thalassemia, confirmed liver disease or kidney disorder (eGFR < 60 mL/min) and recent surgery or blood transfusion (within the last three months) were also implemented as exclusion conditions.

Four questionnaires were used for evaluation of the symptoms: modified Medical Research Council(mMRC), COPD Assessment Test (CAT), Clinical COPD Questionnaire 24 Hour Version (CCQ24) and Clinical COPD Questionnaire 7 Day Version (CCQ7) [23] [27]. The data regarding the number of AECOPD in the past year, smoking status, and dietary habits were collected via additional questionnaire.

### Laboratory investigations

Phlebotomy was performed in the morning, after 12 h of overnight fasting.

Arterial blood samples were collected in heparinized syringes and blood gas analyses were performed on GEM Premier 3500 analyser (Werfen, Lexington, MA, USA) without any delay.

K_2_-EDTA anticoagulated blood samples were used to determine complete blood counts (CBC) with reticulocyte absolute number (RET#), reticulocyte relative number (RET%) including also mean reticulocyte volume (MRV) and immature reticulocyte fraction (IRF). All haematological parameters were obtained within 2 h after sampling on DxH 800 haematology analyser by Beckman Coulter Diagnostics Inc, USA.

The routine biochemistry and immunochemistry tests were performed on blood samples collected in serum tubes without additives. After centrifugation at 1500 g for 10 minutes, serum samples were aliquoted and all aliquots were kept at -80°C until assayed.

Biochemistry analyser AU480 (Beckman Coulter Diagnostic Inc, USA) was used for analyses of serum iron, transferrin, unsaturated iron biding capacity (UIBC) and C-reactive protein (CRP), transferrin and ceruloplasmin. Total iron biding capacity (TIBC) was calculated as the sum of iron and UIBC while transferrin saturation (TSAT) was calculated using the formula TSAT (%) = serum iron/TIBC x 100.

Access 2 immunochemistry analyser (Beckman Coulter Inc, USA) was used for immunochemistry analyses of ferritin, folate, vitamin B12, soluble transferrin receptors (sTfR), interleukin 6 (IL-6) and erythropoietin (EPO) while sTfR/log ferritin index was calculated from sTfR (mg/L) and ferritin (μg/L) concentrations.

Quantikine® Hepcidin Immunoassay (R&D Systems, Inc, MN, USA) with sensitivity of 3.81 pg/mL was used for determination of hepcidin concentrations in serum following manufacturer’s instructions.

Na-citrate anticoagulated blood samples were used for genetic analysis. QiAamp DNA Blood Mini kit (Qiagen Inc., Germantown, Maryland, USA) was used for DNA extraction. Genotypization of TMPRSS6 rs855791 polymorphism, (p.A736V variant) was performed using TaqMan predesigned SNP assays (Thermo Fisher Scienti c Inc., Waltham, MA, USA) on Applied Biosystems 7500 Real-Time PCR instrument (Thermo Fisher Scienti c Inc., Waltham, MA, USA). In the DNA molecule polymorphic site is c.2207 G>A, so obtained genotypes are marked as G/G, A/G and A/A, which corresponds to the usual marks AA, AV and VV at protein level.

### Statistical analysis

IBM Statistical Package for Social Sciences (SPSS) version 20.0 was used for statistical analyses. Kolmogorov–Smirnov test was used for normal distribution evaluation of continuous variables. Variables are presented as mean ± standard deviation or as median (interquartile range, IQR) if normally distributed or non-normally distributed respectively. The relationships between categorical variables were assessed using the Chi-square test. Independent sample t test and Mann–Whitney U test were used for testing the differences between the groups for normally distributed and non-normally distributed variables respectively. The exact Chi-Square test was employed for assessment of deviation of the genotype prevalence in the investigated groups from the assumptions of Hardy−Weinberg Equilibrium. Receiver operating characteristics (ROC) curve analysis was employed for assessment of diagnostic performances of iron status parameters to discriminate between wild-type (WT) genotype and homozygote and heterozygote (HH) genotypes. The optimal ratio of specificity and sensitivity provided the optimal cutoff value. A p- value <0.05 was considered statistically significant.

## Results

The frequency distribution of TMPRSS6 A736V genotypes did not differ between the COPD patient group and control group (Table 1). Frequencies of TMPRSS6 A736V genotypes in both groups were consistent with Hardy–Weinberg equilibrium (Table 1). The two groups also did not show significant differences in gender representation and age of patients.

**Table 1:** Demographic and frequency distribution of TMPRSS6 A736V genotypes in COPD patient and control groups. A736V, substitution of alanine (A) with valine (V) at the position 736; COPD, chronic obstructive pulmonary disease; TMPRSS6, transmembrane protease serine 6, p<0.05 – statistically significant difference

	COPD group (n=107)	Control group (n=192)	p value
Male, n (%)	59 (55.1)	84 (43.8)	0.070
Age, years	67.0 (62.0–70.0)	66.0 (61.0–69.0)	0.617
TMPRSS6 A736V genotype			
Wild type G/G (p.AA), n (%)	33 (30.8)	65 (33.9)	0.791
Homozygous A/A (p.VV), n (%)	19 (17.8)	36 (18.8)	
Heterozygous A/G (p.AV), n (%)	55 (51.4)	91 (47.4)	
Hardy-Weinberg Equilibrium, χ^2^ (p)	0.285 (0.867)	0.168 (0.920)	/

Clinicians have confirmed stable COPD in 94 out of 107 patients (87.9%) which were divided in two groups according to detected genotype: 29 patients (30.9%) with wild-type genotype were classified in one group (WT group) while 65 patients (69.1%) with either homozygous or heterozygous genotype were in the second group (HH group).

The demographic and clinically relevant information of the groups are shown in Table 2. The gender representation and age of patients showed no difference between the groups. A Chi-square test for independence did not show significant association between gender and genotype χ^2^(1, n=94) =3.08, p=0.079, phi=-0.2. Body mass index (BMI), smoking status and cumulative effect of smoking showed statistically insignificant differences between the groups, although patients in HH group tend to smoke less. Pulmonary function and blood gas analyses showed neither statistical nor clinical significance between the groups (Table 2).

**Table 2:** Stable COPD patients’ demographic and clinical characteristics. BMI, body mass index; CAT, COPD assessment test; CCQ24, Clinical COPD Questionnaire 24 h Version; CCQ7, Clinical COPD Questionnaire 7 day Version; COPD, chronic obstructive pulmonary disease; FEV_1_, forced expiratory volume in first second; FVC, forced vital capacity; GOLD, Global initiative for chronic obstructive lung disease; HH, homozygous or heterozygous genotype; mMRC, Modified Medical Research Council Questionnaire; pCO_2_, partial pressure of arterial carbon dioxide; pO_2_, partial pressure of arterial oxygen; SO_2c_-oxygen saturation calculated; WT, wild-type genotype; * -mean±SD; # -median (IQR); p-value <0.05 statistically significant difference

	WT group (29)	HH group (65)	p value
Male, n (%)	11 (37.9)	39 (60.0)	0.079
Age, years	66.0 (60.5–71.0)#	67.0 (63.5–71.0)#	0.608
BMI kg/m^2^	25.6 (22.8–34.0)#	26.1 (23.3–29.8)#	0.997
Smoking status			0.635
Ex-smoker, n (%)	18 (62.1)	45 (69.2)	
Smoker, n (%)	11 (37.9)	20 (30.8)	
Pack- years	40.0 (29.0–46.5)#	35.0 (27.5–47.0)#	0.272
COPD Duration	8.0 (4.0–12.0)#	10.0 (5.0–15.0)#	0.115
GOLD Classification			/
GOLD 1, n (%)	1 (3.5)	7 (10.8)	
GOLD 2, n (%)	18 (62.1)	22 (33.8)	
GOLD 3, n (%)	7 (24.1)	25 (38.5)	
GOLD 4, n (%)	3 (10.3)	11 (16.9)	
FEV1			0.180
FEV_1_ ≤ 50, n (%)	11 (37.9)	36 (55.4)	
FEV_1_ > 50, n (%)	18 (62.1)	29 (44.6)	
Pulmonary Function			
FEV_1_	53.1 ± 18.3*	50.0 ± 20.3*	0.475
FVC	89.7 ± 16.7*	90.3 ± 19.8*	0.899
FEV_1_/FVC	48.8 ± 10.4*	43.9 ± 12.8*	0.076
Blood gas analysis			
pH	7.43 (7.39–7.45)#	7.42 (7.39–7.44)#	0.508
pCO_2_ (kPa)	5.5 (5.0–6.4)#	5.5 (4.9–6.0)#	0.422
pO_2_ (kPa)	9.0 (8.1–10.1)#	9.0 (8.5–9.9)#	0.851
Lactate (mmol/L)	1.60 (1.18–1.80)#	1.60 (1.20–1.90)#	0.987
SO_2c_ (%)	94.0 (90.0–95.3)#	93.0 (90.5–95.0)#	0.895
Questionnaires			
mMRC	2.0 (1.0–2.0)#	2.0 (1.0–3.0)#	0.433
CAT	13.0 (10.0–18.5)#	17.0 (11.0–21.0)#	0.267
CCQ (24h)	17.0 (12.0–23.0)#	16.0 (12.0–22.5)#	0.700
CCQ (7d)	18.0 (11.0–23.5)#	19.0 (14.0–26.0)#	0.466

The results of four questionnaires used for symptom assessment revealed that most of the patients in both groups had mild symptoms and were classified as either GOLD 2 or GOLD 3 [29] [30] [31].

Parameters of iron status showed neither statistically nor clinically significant difference between the groups. Complete blood count (CBC) parameters were with their respective reference values with substantial overlap of the values between the two groups (Table 3). The same trend was noticeable across biochemistry and immunochemistry tests. Markers of inflammation WBC count, CRP and IL-6 were also within their respective reference ranges and did not differ statistically or clinically between the studied groups.

**Table 3:** Parameters of iron status and inflammation. CRP, C- reactive protein; EPO, erythropoietin; HH, homozygous or heterozygous genotype; IL-6, interleukin 6; IRF, immature reticulocyte fraction; MCH, mean cell haemoglobin; MCHC, mean cell haemoglobin corpuscular; MCV, mean cellular volume; MRV, mean reticulocyte volume; RBC, red blood cells; RDW, red cell distribution width; RET, reticulocytes; sTfR, soluble transferrin receptor; TIBC, total iron binding capacity; TSAT, transferrin saturation; WBC, white blood cells; WT, wild-type genotype; * -mean±SD; # -median (IQR); p- value <0.05 statistically significant difference

	WT group (29)	HH group (65)	p value
WBC (10^9^/L)	7.2 ± 1.4*	7.0 ± 1.4*	0.528
RBC (10^12^/L)	4.7 ± 0.4*	4.8 ± 0.5*	0.851
Haemoglobin (g/L)	145.2 ± 10.8*	145.9 ± 12.8*	0.784
Haematocrit (L/L)	0.438 ± 0.032*	0.440 ± 0.038*	0.899
MCV (fL)	92.9 ± 5.0*	92.7 ± 5.0*	0.881
MCH (pg)	30.7 ± 1.9*	30.8 ± 1.8*	0.903
MCHC (g/L)	331 ± 7*	332 ± 7*	0.478
RDW (%)	14.0 (13.5–14.7)#	14.0 (13.3–14.8)#	0.777
RET (%)	1.01 ± 0.30*	1.04 ± 0.34*	0.651
RET (10^9^/L)	44.4 (35.7–51.4)#	45.9 (39.7–57.8)#	0.451
MRV (fL)	116.9 ± 7.8*	115.1 ± 8.5*	0.337
IRF	0.40 ± 0.06*	0.40 ± 0.07*	0.660
Iron (μmol/L)	17.8 ± 5.5*	17.7 ± 6.0*	0.942
TIBC (μmol/L)	60.8 ± 8.6*	61.2 ± 9.5*	0.821
TSAT (%)	30.0 ± 11.3*	29.9 ± 11.6*	0.967
Transferrin (g/L)	2.55 ± 0.41*	2.58 ± 0.45*	0.750
Ceruloplasmin (mg/L)	301.9 ± 52.1*	296.4 ± 48.5*	0.624
Ferritin (μg/L)	68.6 (47.5–111.5)#	68.4 (40.4–130.9)#	0.864
EPO (mIU/mL)	9.9 (6.4– 13.7)#	12.0 (8.2–15.2)#	0.119
Folate (nmol/L)	12.8 (8.9–15.2)#	14.6 (10.9–18.2)#	0.077
Vitamin B12 (pmol/L)	188 (157–254)#	207 (153–291)#	0.474
sTfR (mg/L)	1.51 (1.21–1.91)#	1.55 (1.35–1.88)#	0.386
sTfR (mg)/log ferritin index	0.79 (0.59–1.10)#	0.82 (0.73–1.05)#	0.415
Hepcidin (ng/mL)	29.4 (17.9–36.5)#	26.9 (15.7–35.1)#	0.499
CRP (mg/L)	4.2 (1.9–5.5)#	3.7 (1.7–5.1)#	0.314
IL-6 (pg/mL)	3.4 (2.5–4.4)#	4.3 (2.6–5.1)#	0.174

**Figure 1:**
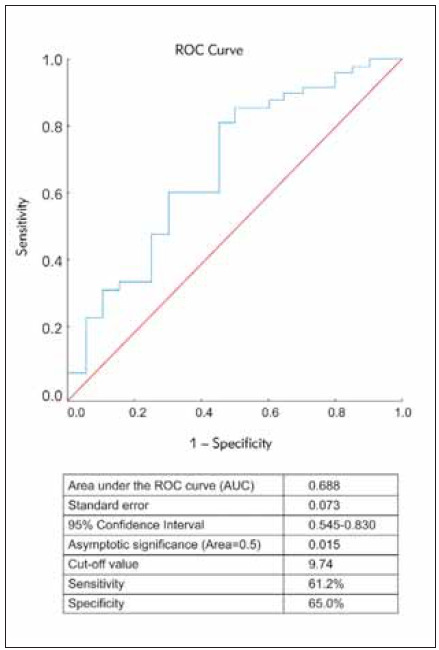
Receiver operating characteristic curve of EPO ability to discriminate between wild-type and mutation genotypes of TMPRSS6.

Laboratory investigations revealed that 3 patients in WT group (10.3%) and 8 patients (12.3%) in HH group had subclinical deficiency of vitamin B12 as per used definition: vitamin B12 concentration ≤133 pmol/L [32]. Additionally, 6 patients (20.7%) in WT group were found to be hypoxemic (employed definition of hypoxemia was pO_2_ < 7.99 kPa). Hypoxemia was also detected in 9 patients (13.8%) in HH group among which there was one patient with vitamin B12 deficiency. Hypoxemia promotes hepcidin decrease which enables supply of sufficient iron for erythropoiesis [2] [33]. Chronic hypoxemia as well as smoking habit in COPD patients is associated with secondary polycythaemia which can affect anaemia recognition in these patients [34]. Several studies demonstrated that vitamin B12 deficiency has been associated with poor iron utilisation in patients with pernicious anaemia and consequently with iron deficiency (ID) camouflage. In order to avoid non-COPD causes of ID, we examined iron status parameters after the exclusion of those patients [9] [35]. The exclusion of these patients did not affect demographic structures of the investigated groups in regard to sex, age and smoking status. Furthermore, pulmonary function, blood gas analyses as well as questionnaires were comparable between the groups, without significant differences statistically or clinically. After the exclusion of this subgroup of patients, the only parameter of iron status that showed statistically significant difference was EPO: as shown in Table 4, the patients with wild type genotype had significantly lower EPO levels than the patients with either homozygous or heterozygous genotype (p=0.015). To further assess the diagnostic performance of EPO to discriminate between WT and HH groups we applied the ROC curve analysis (Figure 1). The AUC of EPO was 0.688 (95% CI: 0.545–0.830, p=0.015) with the best cut-off of 9.74, sensitivity of 61.2% (95% CI:58.1–64.3) and specificity of 65.0% (95% CI: 61.8–68.3).

**Table 4:** Parameters of iron status and inflammation upon exclusion of patients with vitamin B12 deficiency and/or hypoxia. CRP, C- reactive protein; EPO, erythropoietin; HH, homozygous or heterozygous genotype; IL-6, interleukin 6; IRF, immature reticulocyte fraction; MCH, mean cell haemoglobin; MCHC, mean cell haemoglobin corpuscular; MCV, mean cellular volume; MRV, mean reticulocyte volume; RBC, red blood cells; RDW, red cell distribution width; RET, reticulocytes; sTfR, soluble transferrin receptor; TIBC, total iron binding capacity; TSAT, transferrin saturation; WBC, white blood cells; WT, wild-type genotype; * -mean±SD; # -median (IQR); p- value <0.05 statistically significant difference

	WT group (20)	HH group (49)	p value
WBC (10^9^/L)	7.0 ± 1.3*	7.0 ± 1.5*	0.911
RBC (10^12^/L)	4.7 ± 0.4*	4.7 ± 0.5*	0.725
Haemoglobin (g/L)	144.9 ± 11.2*	144.3 ± 12.0*	0.845
Haematocrit (L/L)	0.439 ± 0.033*	0.436 ± 0.037*	0.751
MCV (fL)	93.8 ± 4.6*	92.4 ± 5.3*	0.298
MCH (pg)	31.0 ± 1.8*	30.6 ± 1.9*	0.465
MCHC (g/L)	330 ± 7*	331 ± 7*	0.606
RDW (%)	14.1 (13.5–14.7)#	14.0 (13.4–14.7)#	0.756
RET (%)	0.94 ± 0.24*	1.03 ± 0.33*	0.260
RET (10^9^/L)	43.5 (34.8–49.0)#	45.9 (40.2–56.9)#	0.159
MRV (fL)	116.8 ± 8.3*	114.6 ± 8.1*	0.323
IRF	0.40 ± 0.06*	0.40 ± 0.07*	0.794
Iron (μmol/L)	17.8 ± 5.4*	16.9 ± 5.9*	0.562
TIBC (μmol/L)	62.9 ± 8.7*	60.8 ± 9.5*	0.406
TSAT (%)	29.0 ± 11.1*	28.8 ± 11.5*	0.953
Transferrin (g/L)	2.64 ± 0.43*	2.56 ± 0.45*	0.544
Ceruloplasmin (mg/L)	307.6 ± 47.4*	292.1 ± 49.4*	0.236
Ferritin (μg/L)	69.0 (56.8–108.7)#	68.4 (36.9–130.9)#	0.643
EPO (mIU/mL)	7.0 (5.5– 12.9)#	11.7 (8.2–15.0)#	0.015
Folate (nmol/L)	12.6 (9.6–14.1)#	14.5 (11.7–18.7)#	0.063
Vitamin B12 (pmol/L)	188 (159–242)#	213 (165–290)#	0.179
sTfR (mg/L)	1.40 (1.20–1.80)#	1.49 (1.34–1.82)#	0.275
sTfR (mg)/log ferritin index	0.76 (0.58–1.06)#	0.80 (0.73–0.98)#	0.308
Hepcidin (ng/mL)	30.7 (19.6–35.5)#	27.1 (15.7–35.3)#	0.368
CRP (mg/L)	4.7 (2.5–5.7)#	3.2 (1.7–5.2)#	0.075
IL-6 (pg/mL)	3.3 (2.4–3.9)#	3.9 (2.6–5.8)#	0.231

## Discussion

In this study we assessed the prevalence of SNP rs855791 TMPRSS6 A736V polymorphism among COPD patients and evaluated whether this polymorphism influences iron status parameters including hepcidin in stable COPD patients. Our results showed that iron status parameters do not differ between wild-type genotype carriers and either homozygous or heterozygous genotype carriers. However, underlying conditions such as hypoxia and/or vitamin B12 (sub)clinical deficiency affect iron homeostasis. Thus, exclusion of patients with these conditions revealed statistically significant differences in EPO levels between wild-type genotype carriers and either homozygous or heterozygous genotype carriers.

Iron homeostasis disturbances are involved in several acute and chronic lung diseases [36] [37] [38]. Several GWAS have emphasised that abnormal iron metabolism could have an important role in COPD development. Furthermore, iron homeostasis disturbances as well as iron accumulation within cells in the clinical setting of COPD is rather systemic than locally (in lungs) observed phenomenon. Hence, different indicators of iron metabolism (i.e., genetic, molecular, biochemical) may provide relevant biomarkers of COPD development and progression [5].

To the best of our knowledge this is the first time that TMPRSS6 genotype prevalence was assessed in COPD patient population. Our results revealed that the frequency of TMPRSS6 genotypes in COPD patient population do not differ from those in general population in Serbia (Table 1), and that they are consistent with Hardy-Weinberg equilibrium. Similarly, Dopsaj et al. [39] have assessed the prevalence of TMPRSS6 genotypes and alleles in nephrological patients with end-stage renal disease in Serbian population showing that there is statistically insignificant difference in frequency distribution of the genotypes in patients with iron deficiency anaemia compared topatients with anaemia of chronic disease.

Stable COPD was confirmed in 94 patients by a clinician with most of them being classified as GOLD 2 or GOLD 3. The results of employed questionnaires used for symptom assessment revealed mild symptoms of the disease in the majority of patients as shown in Table 2 [29] [30] [31]. None of the four questionnaires, including the Serbian version of CAT validated by Milenković et al. [25] showed statistical or clinical differences between the groups. However, patients with wild-type genotype (WT group) tend to have lower scores of CAT test than patients with either homozygous or heterozygous genotypes (HH group) with medians being 13.0 and 17.0 for the WT and HH group respectively (Table 2).

Regarding iron status parameters, we observed neither statistical nor clinical difference between the patients with wild-type genotype and patients with either homozygous or heterozygous genotype. The CBC parameters were all within their respective reference ranges with significant overlap of the values between the groups. As shown in Table 3, we obtained almost identical values of RBC, haemoglobin as well as of all other erythrocyte indices and biomarkers of erythropoiesis in the studied groups.

The same results were obtained even after exclusion of the subgroup of patients with detected (sub)clinical vitamin B12 deficiency and/or hypoxia. Chambers et al. [18] reported the association between rs855791 in TMPRSS6 and the concentration of haemoglobin 1.3 g/L (95% CI: 0.9–1.7) lower per copy of allele A (P = 1.6×10^−13^) in their genome-wide study in Europeans and Indian Asians. Similarly, Benyamin et al. [19] in their genome-wide association study demonstrated the role of TMPRSS6 in iron homeostasis and normal erythropoiesis. They identified a significant association of SNPs in TMPRSS6 with several iron status parameters: serum iron (rs855791, combined P=1.5×10^−20^), transferrin saturation (combined P=2.2×10^−23^), and mean cell volume of erythrocytes (MCV, combined P=1.1×10^−10^). Furthermore, the authors showed relevant data on association of SNP in TMPRSS6 with blood haemoglobin concentration (combined P=5.3×10^−7^). Potential origin of these differences may be found in clinical and demographic features of investigated groups. Both Chamber et al. [18] and Benyamin et al. [19] performed genome-wide association studies whereas our cohort consisted of stable COPD patients with low-grade inflammation.

Tandara et al. [11] reported higher values of inflammatory markers in stable COPD patients suggestive of persistent low-grade inflammation. The results of our study are in agreement with these findings: although there was neither statistical nor clinical difference in IL-6 values between the groups, the medians of 3.4 and 4.3 for IL-6 in WT and HH group respectively suggest the low-grade inflammation and comparable to those obtained by Tandara et al. [11]. This increased level of IL-6 could be the cause of increased hepcidin synthesis in the liver or in the lungs where it is synthesised by alveolar macrophages stimulated by lipopolysaccharide [11].

The results of biochemistry and immunochemistry tests of iron status used in this study were very similar and without clinical or statistical differences between the investigated groups. Performed laboratory tests revealed a subgroup of stable COPD patients with (sub)clinical deficiency of vitamin B12 and/or hypoxia. Those conditions can alter iron status: hypoxia decreases hepcidin levels [2] [33] and vitamin B12 deficiency has been associated with masking iron deficiency [35]. To eliminate possible effects of those conditions on iron status parameters, this subgroup of patients was excluded and the results were re-assessed. Following exclusion of this sub-group of patients, total number of stable COPD patients reached 69 out of which 20 patients (29.0%) were with wild-type genotype and 49 patients (71.0%) with either homozygous or heterozygous genotype. Nevertheless, the frequency distribution of TMPRSS6 A736V was not affected significantly. Following the exclusion of this subgroup of patients, we obtained statistically significant difference in EPO values: the patients with mutation of TMPRSS6 (either homozygous or heterozygous) had significantly higher results of EPO than patients with wild type genotype. The assessment of diagnostic usefulness of EPO to discriminate between those two groups provided us with AUC of 0.688. The best cut-off value was 9.74 mIU/mL with sensitivity of 61.2% and specificity of 65.0%. Increased levels of in ammatory markers and notably IL-6 in the stable phase, implies low-grade in ammation in both patient groups. The elevation of inflammatory cytokines (i.e., IL-6) could negatively affect erythropoiesis via reduced production of erythropoietin but also via resistance to EPO−with increased EPO synthesis but diminished erythropoietic response of the bone marrow to it. Furthermore, elevated IL-6 could be responsible forhepcidin-induced reduced extracellular iron availability [3] [9] [11] [14].

Although statistically insignificant differences in IL-6, hepcidin and EPO levels were obtained between the groups, we observed slightly increased levels of IL-6 in the HH patient group.

Since IL-6 induces hepcidin and p.736 V allele has been associated with increased hepcidin and consequently decrease in serum iron in general population (caused by inefficient hepcidin transcription inhibition), we expected to observe higher hepcidin levels in group of patients with either homozygous or heterozygous genotype (HH group) [2] [16] [20]. Interestingly we observed slightly lower levels of hepcidin in the HH group but also higher EPO levels in the same group. In the clinical setting of COPD, EPO levels could be decreased because of the presence of inflammatory cytokines, but also increased as a normal response to tissue hypoxia [9] [12]. When accelerated erythropoiesis occurs, increased EPO levels are associated with suppression of hepcidin synthesis and consequently increase in iron necessary for erythropoiesis [40]. The results of the study performed by Peng et al. [41] have demonstrated that EPO would be effective in restoring normal haematological parameters to IRIDA patients carrying TMPRSS6 mutations. The results of present study are in line with these findings: COPD patients with either homozygous or heterozygous genotype had statistically significant higher values of EPO (Table 4) which could be the cause of normal (within their respective reference ranges) haematological parameters in this patient group.

It was shown that in hypoxia, trauma or in ammation, many tissues produce EPO which has important roles in tissue protection and restoration. EPO is an important regulatory factor that helps maintain immune homeostasis: EPO (and its derivatives) tend to shift macrophages from M1 to M2 phenotype to down-regulate inflammatory reactions. Also, it directly supressesses lymphocytes and influences the balance of T helper cells subsets [42]. In the clinical context of COPD with low grade inflammation, these roles of EPO could be the mechanism of down-regulation of inflammation. With its proven role in erythropoiesis, this anti-inflammatory effect of EPO could contribute to iron homeostasis.

Gammella et al. [40] demonstrated that erythropoietin inhibits hepcidin expression indirectly: direct binding of EPO to its receptors in the liver is not mandatory for the hepcidin suppression to occur. The authors suggest that EPO stimulates the synthesis of the erythroid regulator erythroferrone in erythroblasts which then inhibits hepcidin synthesis.

Although chronic inflammation is thought to be the main reason for iron homeostasis disruption in the clinical setting of COPD, several other factors should be considered and addressed. Nunes et al. [9] summarised possible mechanisms leading to anaemia in COPD. They are roughly classified into inflammationbased and non-inflammation-based mechanisms. Nevertheless, most probably inflammation together with several other factors simultaneously modulate the development of anaemia in COPD patients [9]. In the clinical context of stable COPD with low-grade inflammation and normal serum iron, patients with either homozygous or heterozygous TMPRSS6 genotype could have increased levels of EPO as a corrective mechanism to control hepcidin. The results of different studies have confirmed that hepcidin regulation channels are not functionally completelyindependent of one another. Thus, hepcidin concentrations are regulated by the relative strengths of the individual and (sometimes) antagonistic regulators and signals [11].

The limitations of our study include a relatively low number of enrolled COPD patients even thoughthey are recruited in two healthcare centres. Most of the patients were classified as GOLD 2 or GOLD 3. As per electronic record, oxygen supplementation was not used by the COPD patients, however we cannot exclude the possibility that some of the recruited patients used home oxygen therapy. Standardisation of hepcidin assays is still not achieved: reference material, reference method as well as commutable calibrator are still debatable. These reflect significant variation in absolute hepcidin levels between the assays [22]. In the present study, hepcidin levels were measured by ELISA test which measures all isoforms of hepcidin rather than bioactive hepcidin-25 isoform. Hepcidin ELISA assays, and especially the ones which measure total hepcidin, tend to give higher results compared to mass spectrometry due to crossreactivity with other isoforms [43]. Nevertheless, ELISA is the method of first choice due to low limit of detection, high throughput and low costs. For disorders with increased concentrations of hepcidin isoforms e.g., chronic kidney disease, mass spectrometry is preferred [44].

## Conclusion

The results of several genome-wide association studies (GWAS) have emphasised the possible role of iron homeostasis disruption in development and progression of COPD. Hepcidin has been recognized as a key regulator of systemic iron homeostasis, which regulates absorption of iron via degradation of ferroportin in enterocytes but also in macrophages where iron-recycling occurs [45].

The SNP rs855791 has been linked to iron deficiency anaemia, increased hepcidin, decreased serum iron and reduced haemoglobin indices. Our results demonstrate that in the stable COPD patients with low-grade inflammation, iron status parameters do not differ between wild-type genotype carriers and either homozygous or heterozygous genotype carriers. Upon exclusion of subgroup of patients with (sub)clinical vitamin B12 deficiency and/or hypoxia, statistically significant but clinically irrelevant difference in EPO levels occurs between the two groups of patients suggesting that EPO might control hepcidin levels and thus prevents iron homeostasis disruption which includes development of iron deficiency and further anaemia. In the clinical context of COPD with low grade inflammation, EPO’s direct effect on immune cells and down-regulation of inflammatory reactions could contribute to iron homeostasis preservation. Further research is required to confirm our findings in other subgroups of patients with COPD.

## Dodatak

### Conflict of interest statement

All the authors declare that they have no conflict of interest in this work.
